# Academic workload and lifestyle predict emotional well-being among university students in the United Arab Emirates: A cross-sectional study

**DOI:** 10.1371/journal.pone.0347553

**Published:** 2026-04-20

**Authors:** Munawar Farooq, Uffaira Hafeez, Amir Ahmad, Susan Waller, Gabriel Andrade, Arif Alper Cevik, Syed Fahad Javaid

**Affiliations:** 1 Department of Internal Medicine, Emergency Medicine Section, College of Medicine and Health Sciences, United Arab Emirates University, Al Ain, United Arab Emirates; 2 Emergency Department, Tawam Hospital, Al Ain, United Arab Emirates; 3 Department of Information Systems and Security, United Arab Emirates University, Al Ain, United Arab Emirates; 4 Department of Medical Education, College of Medicine and Health Sciences, United Arab Emirates University, Al Ain, United Arab Emirates; 5 Department of Psychology, Ajman University, Ajman, United Arab Emirates; 6 Department of Psychiatry, College of Medicine and Health Sciences, United Arab Emirates University, Al Ain, United Arab Emirates; Prague University of Economics and Business: Vysoka Skola Ekonomicka v Praze, CZECHIA

## Abstract

**Background:**

Stress is a prevalent issue among university students and is linked to adverse academic and emotional outcomes. While research emphasizes the roles of resilience, personality traits, and psychosocial factors, most studies are drawn from North American and European contexts.

**Objectives:**

This is the first study of its kind in the United Arab Emirates (UAE) exploring the relationship between perceived stress, resilience, and personality traits among university students, offering insights into region-specific influences on emotional well-being.

**Methods:**

An online cross-sectional survey was conducted among 168 students from two colleges at the United Arab Emirates University (79% College of Medicine and Health Sciences, 21% College of Information Technology; 72% female). Data were analyzed using descriptive statistics and regression models in R version 4.2.0. Personality traits were assessed using the Ten-Item Personality Inventory, perceived stress was measured with the Perceived Stress Scale, and resilience was evaluated with the Brief Resilience Scale.

**Results:**

The median perceived stress score was 22 (IQR: 17–28), and 30% reported high stress. Multivariable analysis showed that heavier academic workload, financial difficulties, lack of social support, lower physical activity, and poorer academic performance significantly predicted higher perceived stress, whereas resilience and emotional stability were protective.

**Conclusion:**

University students’ perceived stress is closely associated with modifiable factors, including academic workload, social support, resilience, and physical activity. Targeted interventions, such as resilience training, promoting physical activity, optimizing academic schedules, and strengthening support services, are vital to reducing perceived stress and enhancing student well-being.

## Introduction

The psychological and emotional aspects of well-being are increasingly recognized as key components of a productive life [1]. Stress, defined as the body’s reaction to mental, emotional, or physical disruption [2], is a common experience affecting a significant portion of the global population [3–5] and is particularly prevalent among students. In the United States, 33.9% of students reported stress [6], and medical students showed higher stress than gender-matched peers [7]. Studies from the Middle East also document high levels of stress: Chaabane et al. reported high stress in nursing students [8], Cheema et al. found similar results in Qatar [9], Mirza et al. identified significant stress in 38% of Saudi students [7], and a study in the UAE reported stress prevalence at 29% [10].

Student stress has wide-ranging implications, including reduced academic performance [11], impaired professionalism and empathy in medical training [1], and long-term effects on future patient care quality. Stress outcomes are shaped by multiple factors, including gender [12,13], financial strain [14–17], and academic pressures such as frequent examinations [18,19]. Declining performance is both a cause and consequence of stress, increasing susceptibility to depression [11]. Personality traits also contribute: neuroticism consistently predicts poor well-being, while extraversion and conscientiousness are linked to better regulation, satisfaction, and social connectedness [20–23]. High stress levels in university students have been consistently linked with adverse outcomes, including anxiety, depressive symptoms, academic impairment, and increased risk of burnout, underscoring the importance of understanding stress determinants in this population [24–26].

Protective factors such as physical exercise [27,28], voluntary work [29], hobbies [30,31], and strong social support [32,33] reduce stress, while resilience has emerged as a key moderator of stress responses. Resilience is the process of adapting when faced with adversity, trauma, or any other major source of stress [34]. Resilience is negatively associated with perceived stress in nurses and nursing students [35], and evidence from Greece demonstrates its protective effect across genders [36]. Personality traits further influence resilience, with neuroticism, extraversion, openness, and hopelessness identified as predictors [37,38]. Liu et al. showed that resilience moderates the effects of neuroticism and agreeableness, and that rumination exacerbates stress responses [39]. Together, these findings highlight the complex interplay of personality, resilience, and psychosocial factors in shaping student stress experiences.

In the Middle East, additional socio-political, economic, and cultural pressures, including rapid modernization, urbanization, and displacement, intensify these challenges [8]. At the same time, stigma surrounding mental health and the limited applicability of Western models, which often overlook spirituality and the central role of family, further hinder effective interventions [8,40].

Although student stress has been widely studied, far fewer investigations have examined how personality traits shape stress responses in non-Western university settings. Traits such as emotional stability are strong predictors of stress reactivity and negative emotional states in young adults, yet this evidence is drawn almost entirely from Western and East Asian samples [39,41]. Little is known about these associations in the culturally distinct context of the Arab world. Understanding student perceived stress within the UAE context is particularly important, given the unique cultural expectations, competitive academic structures, and strong social and familial influences that shape student well-being. Recent studies from the UAE have demonstrated substantial emotional health concerns among undergraduates, elevated psychological distress, and academic pressures that differ from Western higher-education settings [10,42,43]. Despite this, very limited research has examined how perceived stress, resilience, and personality traits interact in UAE university students. The present study addresses this gap by providing context-specific evidence on individual and academic determinants of perceived stress within a Gulf-region undergraduate population. By incorporating validated personality measures, this study provides new, region-specific evidence on how individual differences influence perceived stress among UAE undergraduates. To our knowledge, this is the first study of its kind in the United Arab Emirates (UAE) investigating a more nuanced interplay between personality, psychosocial factors, and resilience in this region. This study aims to inform culturally sensitive interventions, public health campaigns, and educational strategies that support student well-being.

## Materials and methods

### Study design and setting

This study represents the **baseline cross-sectional phase of an ongoing prospective cohort**. This study was conducted at the United Arab Emirates University (UAEU) and examined perceived stress, resilience, personality traits, and associated academic and psychosocial factors among undergraduate students. The study focused on students from two colleges, the College of Medicine and Health Sciences (CMHS) and the College of Information Technology (CIT). CMHS and CIT were selected because they represent two academically demanding yet structurally distinct programmes within UAEU. Both follow the same academic calendar and assessment framework, admit students through competitive entry, and report some of the highest levels of academic workload and support-service utilization within the university.

### Participant recruitment and eligibility

As the aim of this baseline analysis was exploratory and descriptive, a formal a priori sample size calculation was not required; instead, a naturalistic, full-capture sampling strategy was used. A non-probabilistic opportunistic sample was taken based on availability and willingness to participate. All currently enrolled undergraduate students aged 18 years or older in CMHS and CIT were eligible to participate, and no exclusion criteria were applied. All students who completed the survey during the recruitment window were included. Recruitment occurred between 1 March and 31 July 2024 via university email announcements and student learning management platforms. Participation was voluntary and anonymous.

### Data collection procedure

The participants accessed the survey through a secure Google Forms link. Prior to beginning the survey, participants were presented with an information sheet outlining the purpose of the study, data confidentiality, and their right to withdraw. Electronic informed consent was required to proceed. As the survey link was disseminated through open university channels without individual-level tracking, the number of students who viewed or accessed the invitation could not be determined; therefore, a formal response rate cannot be calculated. The online questionnaire took approximately 5–8 minutes to complete. The questionnaire comprised validated psychometric scales alongside structured items developed for this study. All items in the online survey were mandatory, ensuring a complete dataset. Therefore, no missing data were generated or imputed. Completed responses were stored on encrypted university servers accessible only to the research team.

### Measures

**1. Demographic and academic information**

Participants reported age, gender, nationality, relationship status, accommodation type, and any known physical or mental health conditions. Academic information included year of study and, for CMHS students only, the survey included an item asking about their intended future area of medical practice. This question was not applicable to CIT students and was not used in comparative or regression analyses. The questionnaire was reviewed by two experienced medical educators and piloted to ensure the clarity and directness of the questions. Questions were then refined and rephrased according to the feedback. However, the questionnaire was not culturally validated, and psychometric validity was not formally tested.

Academic workload was assessed using a set of structured questions designed to quantify recent and upcoming academic demands. Students reported the number of assessments completed in the past week, the number of assessments scheduled in the following two weeks, and the number of assignments completed over the preceding two weeks. Parallel items captured anticipated assignments for the upcoming two weeks to reflect short-term academic pressure. This short-term workload measure is consistent with prior research showing that assessment and assignment frequency are key drivers of perceived workload and stress. Pardos et al. demonstrated that the number of assignments explains substantially more variance in perceived workload than traditional semester-based credit metrics [44]. Daily-diary research also indicates that academic stress fluctuates meaningfully over short periods, supporting the validity of short-term indicators [45]. In a study of Malaysian biomedical-science students, weekly assignment frequency and study hours were used to quantify workload and were significantly associated with stress [46]. Together, these findings support our approach of using recent and upcoming assessments and assignments as objective indicators of acute academic workload. Accordingly, these indicators were intended to reflect acute workload exposure within a short observation window rather than cumulative academic intensity across the semester or programme.

Perceived academic standing was measured using a four-level self-rating scale (Outstanding, Above Average, Pass, Borderline/Fail), which allowed us to examine the relationship between academic performance and perceived stress. Recent feedback was assessed using a binary (yes/no) item asking whether the student had received academic feedback, verbal or written, within the previous two weeks.

**2. Personality traits**

Personality traits were evaluated using the Ten-Item Personality Inventory (TIPI), a validated brief measure of the Big Five dimensions: extraversion, agreeableness, conscientiousness, emotional stability, and openness to experiences [38]. The TIPI consists of 10 items, with two items (one positively and one negatively keyed) representing each trait. Responses are rated on a 7-point Likert scale ranging from 1 (“Disagree strongly”) to 7 (“Agree strongly”). Each TIPI trait score was calculated as the mean of two items (one reverse-scored), yielding scores from 1 to 7. Higher scores indicate stronger expression of the respective personality dimension [47].

**3. Perceived stress**

Perceived stress was assessed using the 10-item Perceived Stress Scale (PSS-10), a validated instrument designed to measure the degree to which situations in one’s life are appraised as stressful during the preceding month [48]. Items are rated on a 5-point Likert scale ranging from 0 (“never”) to 4 (“very often”). The PSS-10 total score was derived by summing all 10 items after reverse-scoring four positive items, producing a range of 0–40, with higher scores indicating greater perceived stress. In line with established conventions, scores are commonly interpreted as low perceived stress (0–13), moderate perceived stress (14–26), and high perceived stress (27–40) [48].

**4. Resilience**

Resilience was assessed using the Brief Resilience Scale (BRS), a validated six-item instrument designed to measure an individual’s ability to recover or “bounce back” from stress [49]. Items are rated on a 5-point Likert scale ranging from 1 (“strongly disagree”) to 5 (“strongly agree”). BRS scoring followed the validated method of averaging the six items (three reverse-scored), yielding a total score ranging from 1 to 5, with higher values reflecting greater resilience. Consistent with prior research, scores are commonly interpreted as low resilience (1.00–2.99), normal resilience (3.00–4.30), and high resilience (4.31–5.00) [49].

**5. Lifestyle and psychosocial factors**

Several lifestyle and environmental variables known to influence student well-being were assessed [27–33]. Physical and mental health conditions were collected as binary (yes/no) variables without diagnostic details to preserve confidentiality. Physical activity was reported as hours of exercise during the previous week and categorized as: 0 hours; 0–2 hours; 2–4 hours; or >4 hours.

Students also indicated whether they had engaged in hobbies or leisure activities in the past month (yes/no), participated in volunteering activities (yes/no), or experienced financial hardship during the past month (yes/no). Awareness of institutional support services was assessed using a binary (yes/no) item asking whether students were aware of the counselling, academic support, and well-being services provided by the university. Perceived social support was measured using two self-report items included in the questionnaire:

1. *“Do you feel you had enough social support during the last month?”* (yes/no)
2. *“Do you get enough support from family and friends when needed?”* (5-point Likert: strongly agree to strongly disagree)

For analysis, responses were dichotomised into high perceived support (yes; agree/strongly agree) versus low perceived support (no; neutral, disagree, strongly disagree). Because the questionnaire was designed to be brief and feasible for a short online survey, we used two pragmatic items to capture students’ perceived availability of support in their immediate social environment. Although these items were not intended to replace a validated multidimensional social-support scale, they provided a concise indicator of perceived support; accordingly, findings related to social support should be interpreted as indicative rather than as a comprehensive measurement of the construct.

### Data analysis

Descriptive statistics were computed for all variables. Categorical variables were summarised as frequencies and percentages, and continuous variables were summarised using medians and interquartile ranges (IQRs). Comparisons between CMHS and CIT students were conducted using the Chi-square test or Fisher’s exact test for categorical variables and the Mann-Whitney U test for continuous variables.

To identify predictors of perceived stress, simple linear regressions were first performed. Variables with p < 0.50 were entered into a multivariable linear regression model adjusting for all five TIPI personality traits. Results are reported as beta coefficients (β) with 95% confidence intervals. The regression outputs were inspected for indications of multicollinearity, such as unstable coefficients or inflated standard errors, and no concerning patterns were observed. All statistical analyses were conducted using R version 4.2.0. All procedures were pre-specified before data collection to minimize bias, and the analytical approach was determined prior to inspection of the dataset.

### Ethical considerations

This study was approved by the UAE University Social Sciences Research Ethics Committee (Approval No. ERSC_2023_3267). All procedures conformed to the ethical standards of the Helsinki Declaration (1975, revised 2008). Participants received an information sheet explaining the study and data security procedures and provided informed electronic consent prior to completing the survey. Participation was voluntary and anonymous.

## Results

A total of 168 participants took part in the study, with no exclusions applied. The majority of the participants were from the CMHS (79%). Most respondents were female (72%), with a median age of 20 years (IQR: 18–21). The median perceived stress score was 22 (IQR: 17–28), with over half (54%) falling under moderate perceived stress levels. Table 1 summarizes core participant characteristics, while the full breakdown, including year of study, relationship status, and detailed workload variables, is provided in Supplementary Table 1 (S1 Table).

**Table 1:** Core Participant Characteristics.

Characteristic	N (%) / Summary
College	CMHS: 132 (79%), CIT: 36 (21%)
Gender	Male: 47 (28%), Female: 121 (72%)
Age (years)	Median 20 (IQR 18–21)
Nationality	Emirati: 164 (98%), Non-Emirati: 4 (2.4%)
Accommodation	Hostel: 107 (64%), Private/Home: 61 (36%)
Physical health condition	32 (19%)
Mental health condition	24 (14%)
Study hours	<3 h: 33 (20%), 3–6 h: 72 (43%), 6–9 h: 25 (15%), 9–12 h: 12 (7.1%),>12 h: 26 (15%)
Academic performance	Outstanding: 29 (17%), Above average: 81 (48%), Pass: 49 (29%), Borderline/Fail: 9 (5.4%)
Physical activity during the previous week	>4 h: 12 (7.1%), 2–4 h: 22 (13%), 0–2 h: 66 (39%), 0 h: 68 (40%)
Hobbies	Yes: 99 (59%), No: 69 (41%)
Financial hardship	Yes: 44 (26%), No: 124 (74%)
Social support	High: 84 (50%), Low: 84 (50%)
Resilience (BRS)	Median 3.17 (IQR 2.67–3.50)
Stress category (PSS)	Median 22 (IQR 17–28). Low: 26 (15%), Moderate: 91 (54%), High: 51 (30%)

CMHS = College of Medicine and Health Sciences; CIT = College of Information Technology; BRS = Brief Resilience Scale; PSS = Perceived Stress Scale.

Significant differences were found between the two colleges. More CIT students were male (47% vs 23%, p = 0.004) and reported a heavier academic workload, with a greater percentage having more than three assessments in the last week (44% vs 14%) and in the upcoming two weeks (47% vs 17%) (p < 0.001 for both). A significantly higher proportion of CIT students completed ≥3 assignments in the past two weeks (56% vs 11%) and expected the same in the next two weeks (54% vs 17%) compared to CMHS students (p < 0.001 for both). Fewer CIT students reported receiving feedback (17% vs 44%, p = 0.003) and awareness of university support services (39% vs 71%, p < 0.001). Although the year of study differed between CMHS and CIT students (p = 0.003), it was not associated with perceived stress in either univariate or multivariable analyses (all p > 0.10) and is therefore not reported in detail. Mean resilience scores were also lower among CIT students (2.94 ± 0.40) than CMHS students (3.23 ± 0.79, p = 0.012). Table 2 summarises the comparison of demographic, academic, psychosocial, and resilience characteristics between CMHS and CIT students.

**Table 2:** Comparison of characteristics between CMHS and CIT students.

Variable	CMHS (n = 132)	CIT (n = 36)	p-value
Male gender (%)	23	47	0.004
>3 assessments last week (%)	14	44	<0.001
>3 assessments next 2 weeks (%)	17	47	<0.001
>3 assignments last 2 weeks (%)	11	56	<0.001
>3 assignments next 2 weeks (%)	17	54	<0.001
Received feedback (%)	44	17	0.003
Awareness of university services (%)	71	39	<0.001
Resilience (mean ± SD)	3.23 ± 0.79	2.94 ± 0.40	0.012

### Predictors of perceived stress

After adjusting for TIPI scores, several factors were significantly associated with higher perceived stress scores among students. Some gender differences were observed descriptively; however, gender was formally evaluated within the multivariable model. In the adjusted model, gender was not a statistically significant predictor of perceived stress (β = 2.1, 95% CI –0.07 to 4.2, p = 0.058). Increased academic workload was a strong contributor: having 1–3 assessments in the past week increased perceived stress by 2.9 points (p = 0.006), and more than three assessments by 3.4 points (p = 0.007), compared to none. Students with more than three upcoming assessments in the next two weeks had a 3.7-point increase in perceived stress (p = 0.014). Completing 1–3 assignments in the past two weeks increased perceived stress by 3.0 points (p = 0.006), and completing more than three assignments by 3.5 points (p = 0.009), relative to completing none. Students from the College of Information Technology (CIT) reported a 2.9-point higher perceived stress score compared to those from CMHS (p = 0.012).

Lower physical activity was linked to higher perceived stress. Perceived stress scores were 4.2 points higher in students with 0–2 hours of activity (p = 0.049) and 5.3 points higher in inactive students (0 hours; p = 0.014), compared to those with over 4 hours. Not engaging in hobbies over the past month was associated with a 2.5-point increase in perceived stress (p = 0.010), and financial hardship was linked to a 2.9-point increase (p = 0.007).

Academic performance was inversely related to perceived stress: compared with students reporting outstanding or above-average academic performance, perceived stress scores were 5.1 points higher among those reporting pass-level performance (p = 0.010) and 5.9 points higher among those reporting borderline/fail performance (p = 0.010).

Lack of social support increased perceived stress by 3.2 points (p < 0.001); and compared to those who strongly agreed they had family/friend support, perceived stress scores were higher by 2.3 points (agree, p = 0.032), 5.0 (neutral, p < 0.001), 6.4 (disagree, p = 0.001), and 7.4 (strongly disagree, p = 0.003). The academic year was examined but showed no significant differences between CMHS and CIT students and was not associated with perceived stress; therefore, it is not presented in detail.

**Fig 1:**
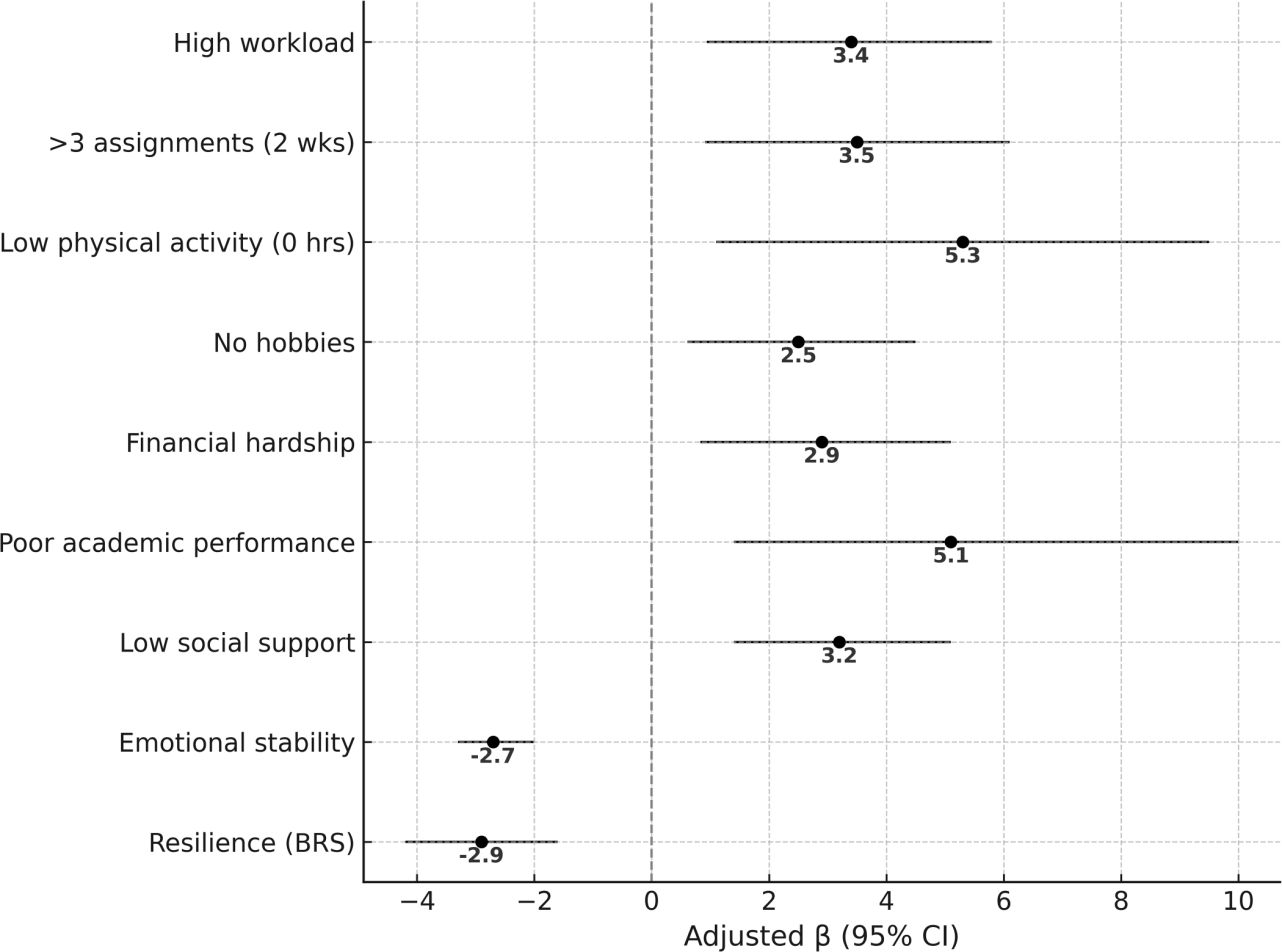
Predictors of perceived stress among university students. **Legend:** Positive values indicate higher perceived stress, while negative values indicate protective effects. Model adjusts for Big Five personality traits (TIPI).

Resilience was protective: Each one-point increase in the Brief Resilience Scale (BRS) was associated with a 2.9-point decrease in perceived stress. Lastly, each unit increase in emotional stability was associated with a 2.7-point decrease in perceived stress (p < 0.001). Fig 1 presents the adjusted regression coefficients (β) with 95% confidence intervals for significant predictors of perceived stress. To facilitate interpretation, the multivariable linear regression model identifying predictors of perceived stress is presented in Table 3. Full regression results, including both crude and adjusted estimates, are provided in Supplementary Table 2 (S2 Table).

**Table 3:** Predictors of Perceived Stress.

Predictor	Reference	Adjusted β (95% CI)	p-value
College (CIT vs CMHS)	CMHS	2.9 (0.65–5.1)	0.012
Gender (Female vs Male)	Male	2.1 (–0.07–4.2)	0.058
Mental health condition	None	1.1 (–1.6–3.8)	0.40
Workload (>3 assessments last week)	≤3 assessments	3.4 (0.94–5.8)	0.007
Assignments >3 (last 2 weeks)	≤3 assignments	3.5 (0.91–6.1)	0.009
Physical activity (0 hours/week)	>0 hours	5.3 (1.1–9.5)	0.014
No hobbies	Hobbies present	2.5 (0.61–4.5)	0.010
Financial hardship	No hardship	2.9 (0.83–5.1)	0.007
Academic performance: Pass	Outstanding/Above average	5.1 (1.4–10)	0.010
Academic performance: Borderline/Fail	Outstanding/Above average	5.9 (1.4–10)	0.010
Low social support	High support	3.2 (1.4–5.1)	<0.001
Emotional stability (per unit ↑)	—	–2.7 (–3.3 to –2.0)	<0.001
Resilience (per unit ↑)	—	–2.9 (–4.2 to –1.6)	<0.001

## Discussion

Our study revealed important insights into the perceived stress levels among university students, highlighting the influence of institutional context, gender, personality traits, and psychosocial factors. In terms of overall perceived stress, approximately 31% of students in our study were classified as highly stressed. Reported prevalence rates in the literature vary considerably. For example, a study from a Nigerian medical college found a high stress prevalence of 59.8% [50], while research in Malaysia reported 17.6% [51]. In the United States, 13.3% of college students in Florida were classified as experiencing severe stress [13], and in Saudi Arabia, the prevalence of high stress was approximately 12.7%. These discrepancies likely reflect differences in academic environments, cultural expectations, available support systems, and the characteristics of the student populations studied.

High levels of perceived stress have been consistently linked to adverse mental-health outcomes, including anxiety, depressive symptoms, emotional exhaustion, and burnout. International evidence reinforces these concerns: multi-country studies of medical students have reported substantial levels of burnout, reduced well-being, and associated risks such as substance use across diverse cultural settings [52–54]. These findings highlight the broader implications of our results and emphasize the importance of early identification and targeted support strategies.

In the univariate analysis, female students reported significantly higher levels of perceived stress, a finding consistent with previous studies [13,55]. The lack of statistically significant gender differences in perceived stress after adjustment suggests that stress interventions should move beyond gender-based generalizations and instead focus on individual student profiles, including personality and resilience levels.

In the univariate analysis, three of the Big Five personality traits: conscientiousness, openness to experience, and emotional stability were significantly associated with lower levels of perceived stress. This aligns with existing literature demonstrating that individual differences in personality are strongly linked to how stress is appraised and managed. Individuals with higher levels of neuroticism (inverse of emotional stability) tend to interpret situations as more stressful and uncontrollable, whereas those higher in conscientiousness, extraversion, and openness are more likely to perceive stressors as manageable [56]. However, in the multivariable model, only emotional stability remained a significant predictor of perceived stress in our sample. Resilience also emerged as an independent protective factor against perceived stress. Students with higher resilience scores reported lower perceived stress, in line with prior studies demonstrating the buffering effect of resilience on stress [57]. Because the primary aim of this baseline analysis was to identify determinants of perceived stress, resilience was examined as a predictor rather than as a separate outcome. Predictors of resilience will be explored in planned longitudinal analyses.

Although CIT students in our sample reported higher perceived stress than CMHS students, this finding should be interpreted in light of how academic workload was operationalized. Much of the existing literature reports higher stress among medical students, often attributed to the cumulative intensity, duration, and clinical demands of medical training. In contrast, our measure captured short-term academic demands, specifically, recent and upcoming assessments and assignments, which represent acute stressors rather than long-term programme load. Prior research has shown that assessment and assignment frequency is a major driver of perceived workload, that academic stress fluctuates meaningfully over short time windows, and is associated with higher stress among university students [44–46]. During the data-collection period, CIT students reported substantially more assessments and assignments within this short-term window, which may explain the higher perceived stress levels observed in this cross-sectional snapshot. This distinction between immediate academic pressure and cumulative curricular intensity suggests that discipline-specific stress patterns may vary depending on the timeframe of workload measurement. Future research should incorporate both acute and long-term workload indicators to better characterize student stress profiles across different programs.

These findings should also be interpreted within the UAE’s cultural and academic context. Undergraduate students in the UAE often navigate strong family expectations, competitive programme structures, and high academic performance standards, all of which may heighten perceived stress and shape coping responses [10,42]. Evidence from recent studies in the UAE has shown substantial emotional health concerns and academic pressures among undergraduates, underscoring the need to consider these contextual factors when interpreting stress and resilience [10,42]. Understanding these region-specific influences is essential for developing culturally attuned well-being interventions and institutional policies. At a global level, multi-country research has similarly documented high levels of burnout, diminished well-being, and academic pressure among medical students across diverse settings, echoing several of the stressors identified in our sample [52–54]. Together, these regional and international patterns reinforce the multifactorial nature of student perceived stress and highlight the importance of interpreting our findings within both local and global academic environments.

A key novel contribution of this study is the finding that personality traits, particularly emotional stability, are significant predictors of perceived stress in UAE undergraduates. This aligns with validated evidence showing that emotional stability and other Big Five dimensions strongly influence stress reactivity and negative emotional states in young adults [56]. However, these associations have not previously been examined in university students in the UAE or the wider Gulf region. Our findings, therefore, provide new, culturally relevant evidence that stable psychological traits play an important role alongside academic and environmental stressors.

Among the examined stressors, perceived support from family and friends showed the strongest association with perceived stress levels. Students who reported lower levels of perceived support experienced progressively higher perceived stress, indicating a clear stepwise relationship. This pattern suggests that as students feel less supported socially, their perceived stress increases correspondingly, underscoring the protective role of strong social support networks in mitigating psychological stress. These findings are consistent with previous studies, which have demonstrated significant negative correlations between perceived stress and social support scales [58]. Similarly, lower levels of physical activity and poorer academic performance showed strong and progressive associations with higher perceived stress. Perceived stress levels increased stepwise with decreasing physical activity, peaking among students who reported no activity. Similarly, students with lower self-reported academic performance experienced progressively higher perceived stress levels. These findings align with a meta-analysis by Wunsch et al [58], which examined the tridirectional relationship among physical activity, stress, and academic performance in university students and found a negative association between physical activity and stress. However, the link between stress and academic performance remained inconclusive.

In contrast, a study among dental undergraduate students found that students with higher GPAs reported lower perceived stress levels, highlighting the potential buffering effect of academic achievement on perceived stress. Academic load, specifically assessments in the past week and the upcoming two weeks, along with assignments in the past two weeks, is a significant stressor in our study. This finding is supported by several studies [8,59,60] that have similarly identified increased academic workload as a key contributor to student stress. This finding that heavier workloads and frequent assessments predict higher perceived stress underscores the need for balanced curriculum planning and effective assessment scheduling. Overloading students, especially without adequate support, may be counterproductive and harm both their well-being and academic performance. Financial hardship also emerged as a significant stressor, aligning with literature linking financial strain to student distress [61]. Conversely, engagement in hobbies was protective, associated with lower perceived stress. However, a study in Sri Lankan undergraduates found no link between extracurricular activities and stress, indicating that the impact of such activities may vary [62].

In our study, CIT students reported higher perceived stress levels compared to medical students, which contrasts with much of the literature that finds higher stress among medical students compared to non-medical students [63,64]. The greater academic workload may explain this difference, as both assignments and assessments experienced by CIT students were significantly higher than those of medical students in our sample. Additionally, CIT students had lower resilience scores. Both academic workload and resilience emerged as significant predictors of perceived stress, suggesting that the combination of heavier academic demands and reduced coping resources contributed to the higher perceived stress levels observed among CIT students.

From an educator’s perspective, these findings highlight that perceived stress is multifactorial, shaped not only by academic workload but also by personality traits, resilience, social support, hobbies, and financial pressures. Social support and extracurricular engagement remain protective factors that enhance well-being [51,65]. At the same time, resilience and emotional stability serve as independent buffers against stress, supporting the value of embedding resilience-building and emotional regulation strategies into curricula through workshops, peer mentoring, mindfulness, or counselling services, particularly during high-stress periods such as examinations [26,49]. Universities can act on these insights by incorporating brief skills-based resilience or well-being modules into early-year courses, reviewing assessment timetables to minimize deadline clustering, and improving the visibility and accessibility of counselling and academic support services. Coordinated implementation of these strategies may strengthen coping resources and foster a more supportive learning environment. At the policy level, embedding well-being considerations into academic structures, such as offering more flexible deadlines and ensuring balanced workload distribution, has been shown to reduce stress and enhance engagement [66]. Recognizing these influences enables universities to create academic environments that support students’ emotional well-being while informing decisions on resource allocation for student welfare and proactive mental health initiatives.

This study provides valuable insights into the emotional well-being of university students in the UAE. Nonetheless, several limitations should be acknowledged. The cross-sectional design restricts the ability to draw causal inferences between perceived stress and its associated factors. Given the cross-sectional design, reverse causation is possible; for example, higher perceived stress levels may reduce resilience, meaning the association observed cannot be assumed to be unidirectional. The voluntary, convenience-based recruitment strategy may also introduce sampling bias, as students who chose to participate may differ systematically from those who did not. The study sample, drawn from two colleges within a single institution and comprising predominantly female and medical students, may not be fully representative of the wider university student population. These proportions reflect voluntary participation patterns rather than the underlying distribution of the wider student body. As such, the findings may not fully generalize to other colleges or to more gender-balanced populations. Future phases of the ongoing prospective cohort will incorporate additional faculties to enhance representativeness and improve the generalizability of findings across the wider university population. The reliance on self-reported data also introduces the potential for recall and social desirability bias. Moreover, the timing of data collection may have influenced the findings, as perceived stress levels are known to fluctuate across the academic year and may peak during major examination periods. Finally, although standardized instruments were used, the inclusion of several self-developed questionnaire items introduces additional measurement limitations. In particular, the brief two-item measure of social support may not fully capture the multidimensional nature of social support and therefore warrants cautious interpretation. Despite these constraints, the study makes a meaningful contribution to the discourse on student mental health in Middle Eastern settings and identifies key targets for intervention.

## Conclusions

This study shows that, within this sample of students from two colleges at one UAE university, perceived stress is shaped by a combination of academic workload, personality traits, resilience, and psychosocial factors such as social support and engagement in hobbies. These findings add to the limited regional literature but should be interpreted within the specific institutional and sampling context. Universities with similar student populations and academic demands may wish to implement structured resilience-building initiatives, integrate well-being and study-skills modules early in students’ academic programmes, review assessment timetables to reduce clustering of deadlines, and enhance the visibility and accessibility of counselling and academic support services. Adopting these strategies in a coordinated manner may help mitigate stress, strengthen coping resources, and support students’ emotional well-being. Future research should employ longitudinal and multi-institutional designs to capture fluctuations in perceived stress across the academic cycle and to test the effectiveness of targeted, personality-informed interventions.

## Supporting information

S1 Table Full Participant Characteristics. Participants’ characteristics with a full breakdown, including year of study, relationship status, and detailed workload variables.
(DOCX)

S2 Table Complete Regression Analysis Results. Full regression results, including both crude and adjusted estimates.
(DOCX)
