# Supplementary material for: Academic workload and lifestyle predict emotional well-being among university students in the United Arab Emirates: A cross-sectional study
Source: PLoS One. 2026 Apr 20;21(4):e0347553. doi: 10.1371/journal.pone.0347553 (PMC13095009; doi:10.1371/journal.pone.0347553)
Supplement: S1 Table — Participants’ characteristics with a full breakdown, including year of study, relationship status, and detailed workload variables. (DOCX) [file pone.0347553.s001.docx]

**S1 Table: Full Participant Characteristics**

| **Characteristic** | **Value** |
| --- | --- |
| Age (years), median (IQR) | 20 (18–21) |
| Gender | Male 47 (28%), Female 121 (72%) |
| Nationality | Emirati 164 (98%), Non-Emirati 4 (2.4%) |
| Accommodation | Hostel 107 (64%), Private/Home 61 (36%) |
| Relationship status | Single 165 (98%), Married 2 (1.2%), Divorced 1 (0.6%) |
| Year 1 | 55 (33%) |
| Year 2 | 14 (8.3%) |
| Year 3 | 20 (12%) |
| Year 4 | 52 (31%) |
| Year 5 | 16 (9.5%) |
| Year 6 | 11 (6.5%) |
| Known physical health condition | 32 (19%) |
| Known mental health condition | 24 (14%) |
| Study hours | <3h: 33 (20%), 3–6h: 72 (43%), 6–9h: 25 (15%), 9–12h: 12 (7.1%), >12h: 26 (15%) |
| >3 assessments last week | 34 (20%) |
| >3 assessments next 2 weeks | 39 (23%) |
| >3 assignments last 2 weeks | 34 (20%) |
| >3 assignments next 2 weeks | 38 (23%) |
| Received academic feedback | 63 (38%) |
| Academic performance | Outstanding 29 (17%), Above Avg 81 (48%), Pass 49 (29%), Borderline/Fail 9 (5.4%) |
| Physical activity | 0h: 68 (40%), 0–2h: 60 (36%), 2–4h: 22 (13%), >4h: 9 (5%) |
| Engaged in hobbies | 99 (59%) |
| Volunteering | 23 (14%) |
| Financial hardship | 44 (26%) |
| Awareness of university services | 108 (64%) |
| Social support (high) | 84 (50%) |
| Resilience (BRS) | Median 3.17 (IQR 2.67–3.50) |
| Perceived stress (PSS) | Median 22 (IQR 17–28) |
| High stress (PSS ≥27) | 51 (30%) |
