# Supplementary material for: Academic workload and lifestyle predict emotional well-being among university students in the United Arab Emirates: A cross-sectional study
Source: PLoS One. 2026 Apr 20;21(4):e0347553. doi: 10.1371/journal.pone.0347553 (PMC13095009; doi:10.1371/journal.pone.0347553)
Supplement: S2 Table — Full regression results, including both crude and adjusted estimates. (DOCX) [file pone.0347553.s002.docx]

# S2 Table: Complete Regression Analysis Results

| Predictor | Reference category | Crude β (95% CI) | p-value | Adjusted β (95% CI) | p-value |
| --- | --- | --- | --- | --- | --- |
| College | CMHS | 1.5 (–1.2 to 4.2) | 0.30 | 2.9 (0.65 to 5.1) | 0.012 |
| Gender | Male | 3.3 (0.9 to 5.8) | 0.007 | 2.1 (–0.07 to 4.2) | 0.058 |
| Mental health condition | None | 3.7 (0.57 to 6.8) | 0.021 | 1.1 (–1.6 to 3.8) | 0.40 |
| Workload (>3 assessments last week) | ≤3 assessments | 3.9 (0.93 to 6.9) | 0.010 | 3.4 (0.94 to 5.8) | 0.007 |
| Assignments >3 (last 2 weeks) | ≤3 assignments | 3.2 (0.01 to 6.4) | 0.049 | 3.5 (0.91 to 6.1) | 0.009 |
| Physical activity (0 hours/week) | >0 hours | 7.2 (2.3 to 12) | 0.004 | 5.3 (1.1 to 9.5) | 0.014 |
| No hobbies | Hobbies present | 4.5 (2.3 to 6.6) | <0.001 | 2.5 (0.61 to 4.5) | 0.010 |
| Financial hardship | No hardship | 3.6 (1.1 to 6.1) | 0.005 | 2.9 (0.83 to 5.1) | 0.007 |
| Academic performance: Pass | Outstanding/Above average | 5.7 (2.4 to 8.9) | <0.001 | 5.1 (1.4 to 10) | 0.010 |
| Academic performance: Borderline/Fail | Outstanding/Above average | 6.2 (0.91 to 12) | 0.022 | 5.9 (1.4 to 10) | 0.010 |
| Low social support | High support | 5.1 (3.1 to 7.2) | <0.001 | 3.2 (1.4 to 5.1) | <0.001 |
| Emotional stability (per unit ↑) | — | –2.8 (–3.4 to –2.2) | <0.001 | –2.7 (–3.3 to –2.0) | <0.001 |
| Resilience (per unit ↑) | — | –4.7 (–6.1 to –3.4) | <0.001 | –2.9 (–4.2 to –1.6) | <0.001 |
